# Loss of Corpus-Specific Lipids in Helicobacter pylori-Induced Atrophic Gastritis

**DOI:** 10.1128/mSphere.00826-21

**Published:** 2021-11-24

**Authors:** Aung Soe Lin, Jennifer H. B. Shuman, Ankita Kotnala, Jeff A. Shaw, Amber C. Beckett, Jennifer L. Harvey, Michael Tuck, Beverly R. E. A. Dixon, Michelle L. Reyzer, Holly M. Scott Algood, Kevin L. Schey, M. Blanca Piazuelo, Timothy L. Cover

**Affiliations:** a Department of Pathology, Microbiology and Immunology, Vanderbilt University School of Medicine, Nashville, Tennessee, USA; b Mass Spectrometry Research Center, Vanderbilt University School of Medicine, Nashville, Tennessee, USA; c Department of Biochemistry, Vanderbilt University School of Medicine, Nashville, Tennessee, USA; d Department of Medicine, Vanderbilt University School of Medicine, Nashville, Tennessee, USA; e Veterans Affairs Tennessee Valley Healthcare System, Nashville, Tennessee, USA; University of Michigan-Ann Arbor

**Keywords:** gastric cancer, atrophic gastritis, imaging mass spectrometry, chief cells, parietal cells

## Abstract

Helicobacter pylori colonization of the stomach is a strong risk factor for the development of stomach cancer and peptic ulcer disease. In this study, we tested the hypothesis that H. pylori infection triggers alterations in gastric lipid composition. Mongolian gerbils were experimentally infected with H. pylori for 3 months. Conventional histologic staining revealed mucosal inflammation in stomachs from the H. pylori-infected animals but not in stomachs from uninfected control animals. Atrophic gastritis (a premalignant condition characterized by loss of corpus-specific parietal and chief cells), gastric mucosal hyperplasia, dysplasia, and/or gastric cancer were detected in stomachs from several infected animals. We then used imaging mass spectrometry to analyze the relative abundance and spatial distribution of gastric lipids. We detected ions corresponding to 36 distinct lipids that were differentially abundant when comparing gastric tissues from H. pylori-infected animals with tissues from uninfected animals. Liquid chromatography-tandem mass spectrometry analysis of lipid extracts from homogenized gastric tissues provided additional supportive evidence for the identification of several differentially abundant lipids. Sixteen of the differentially abundant lipids were localized mainly to the gastric corpus in stomachs from uninfected animals and were markedly reduced in abundance in stomachs from H. pylori-infected animals with severe disease (atrophic gastritis and dysplasia or gastric cancer). These findings indicate that H. pylori infection can lead to alterations in gastric lipid composition and constitute a new approach for identifying biomarkers of gastric atrophy and premalignant changes.

**IMPORTANCE** H. pylori colonization of the stomach triggers a cascade of gastric alterations that can potentially culminate in stomach cancer. The molecular alterations that occur in gastric tissue prior to development of stomach cancer are not well understood. We demonstrate here that H. pylori-induced premalignant changes in the stomach are accompanied by extensive alterations in gastric lipid composition. These alterations are predicted to have important functional consequences relevant to H. pylori-host interactions and the pathogenesis of gastric cancer.

## INTRODUCTION

Helicobacter pylori is a Gram-negative bacterial species that colonizes the human stomach. Approximately half of the global population is currently colonized by H. pylori (1). The majority of individuals colonized by H. pylori do not develop adverse effects, but the presence of H. pylori is a risk factor for development of peptic ulcer disease or gastric adenocarcinoma (2–4). H. pylori colonization of the stomach is the strongest known risk factor for gastric cancer, and stomach cancer is the fourth leading cause of cancer mortality worldwide (5).

H. pylori colonization of the human stomach results in a gastric mucosal inflammatory response characterized by infiltration of neutrophils, lymphocytes, and macrophages (6). Subsequently, a series of premalignant changes can arise, including atrophic gastritis, intestinal metaplasia, and dysplasia (7–9). These alterations can potentially culminate in the development of gastric adenocarcinoma. The risk of gastric cancer is influenced by strain-specific properties of H. pylori, human genetic variation, and environmental factors, including diet (10).

Our understanding of the pathogenesis of H. pylori infection has been aided by experimentation using animal models. Mice and Mongolian gerbils are two of the most commonly used models (11–13). H. pylori can colonize the stomach in both of these rodent species, but there are notable differences in the gastric responses. Both mice and Mongolian gerbils can develop gastric mucosal inflammation in response to H. pylori, but gastric inflammation tends to be more severe in gerbils than in mice (11). Under most circumstances, H. pylori-infected wild-type mice do not develop gastric ulcers or gastric cancer (11). In contrast, H. pylori-infected Mongolian gerbils can develop gastric ulcers, hyperplasia, premalignant pathology (parietal and chief cell loss and dysplasia), or gastric cancer (13–19).

To evaluate gastric histology and pathology, the most used method is hematoxylin and eosin (H&E) staining of fixed, paraffin-embedded tissue. In the current study, we also used matrix-assisted laser desorption ionization–imaging mass spectrometry (MALDI-IMS) and liquid chromatography-tandem mass spectrometry (LC-MS/MS) to analyze gastric tissue. Specifically, we tested the hypothesis that H. pylori infection causes alterations in gastric lipids. Untargeted lipidomics based on imaging mass spectrometry allows for the assessment of the spatial distribution of lipid species within tissues (20, 21), and IMS methodology can reliably detect mass-to-charge ratios (*m/z*) corresponding to a wide range of tissue lipids (22). LC-MS/MS methods provide a more comprehensive analysis of lipids present in tissue along with the ability to identify unique lipids. Using these two methods, we identified multiple lipids that change in abundance in gastric tissue in response to H. pylori infection. Importantly, we identified multiple gastric lipids that are localized mainly to the gastric corpus in stomachs from uninfected animals and that are reduced in abundance in stomachs from H. pylori-infected animals exhibiting atrophic gastritis (a premalignant condition characterized by inflammation and loss of chief cells and acid-secreting parietal cells in the gastric corpus). These alterations are predicted to have important functional consequences relevant to H. pylori-host interactions and the pathogenesis of gastric cancer.

## RESULTS

### Gastric inflammation and disease outcomes in response to H. pylori.

Mongolian gerbils were experimentally infected with H. pylori, and other animals from the same cohort were maintained as uninfected controls. Gerbils were euthanized at 3 months postinfection. A longitudinal strip of gastric tissue from each animal was fixed in formalin and paraffin embedded (FFPE) for H&E staining, and an additional strip of tissue was snap frozen using dry ice for subsequent IMS analysis (Fig. S1 in the supplemental material).

10.1128/mSphere.00826-21.2 FIG S1 Processing of stomachs from Mongolian gerbils. After harvesting the stomach, the nonglandular forestomach was removed (red dotted line). Then, the stomach was cut open along the lesser curvature (red dotted line) and laid flat. The gastric tissue was cut into halves (red dotted line), each containing corpus and antrum, and these were used for histology or IMS analysis. Download FIG S1, TIF file, 0.8 MB. Copyright © 2021 Lin et al. 2021 Lin et al. https://creativecommons.org/licenses/by/4.0/ This content is distributed under the terms of the Creative Commons Attribution 4.0 International license.

**FIG 1:**
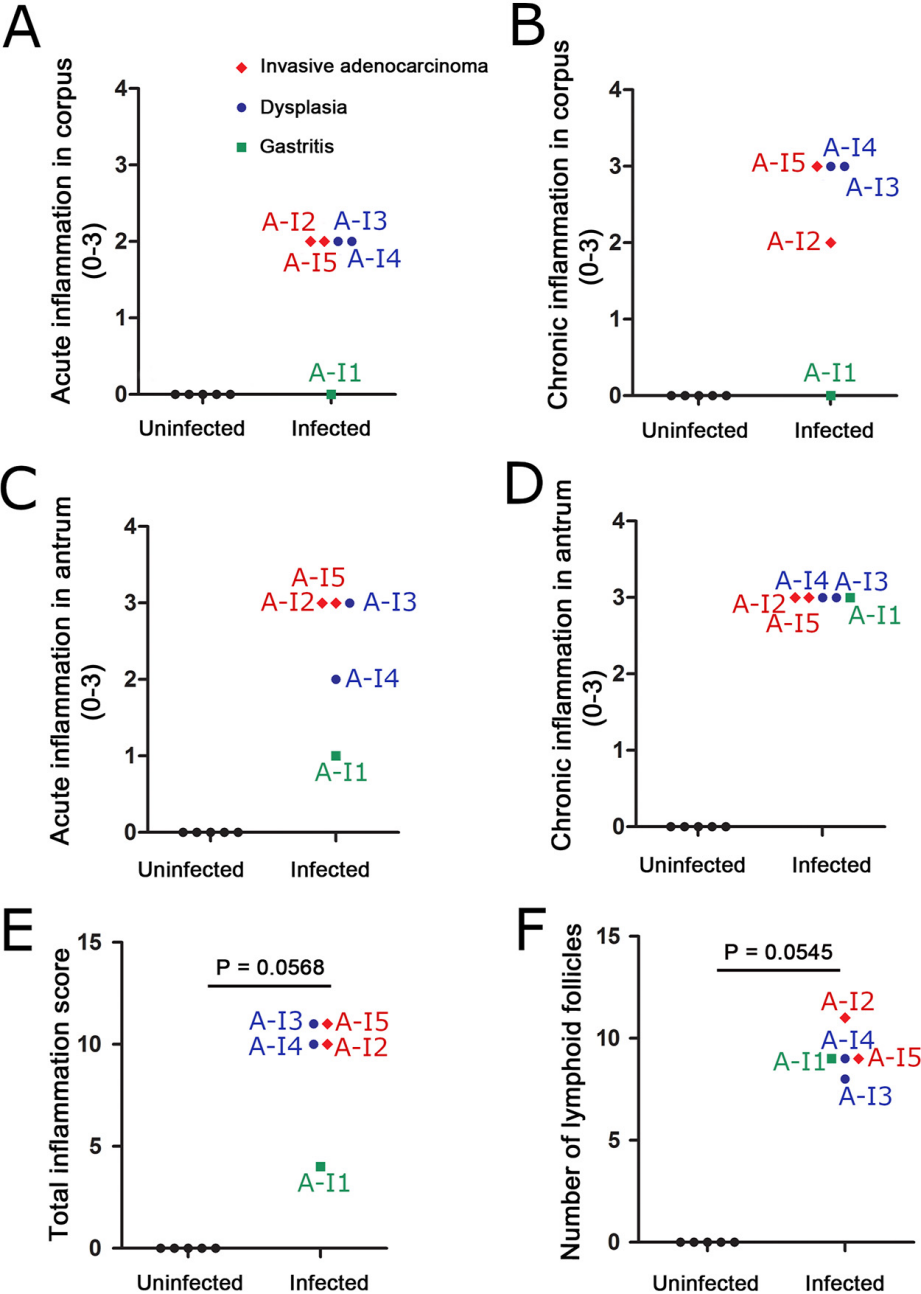
Gastric inflammation in response to Helicobacter pylori infection. (A and B) Acute and chronic inflammation in corpus. (C and D) Acute and chronic inflammation in antrum. (E) Total inflammation scores (combined analysis of corpus and antrum). (F) Number of lymphoid follicles visible in gastric mucosa. Gastric inflammation was scored as described in Materials and Methods. The label “U” and subsequent numbers (1, 2, 3, 4, and 5) represent designations of individual uninfected animals. The label “I” and subsequent numbers (1, 2, 3, 4, and 5) represent designations of individual H. pylori-infected animals, with disease diagnosis indicated by color codes. Uninfected animals are represented by black circles, infected animals with mild disease (gastritis only) by green squares, infected animals with dysplasia by blue circles, and infected animals with invasive adenocarcinoma by red diamonds.

**FIG 2:**
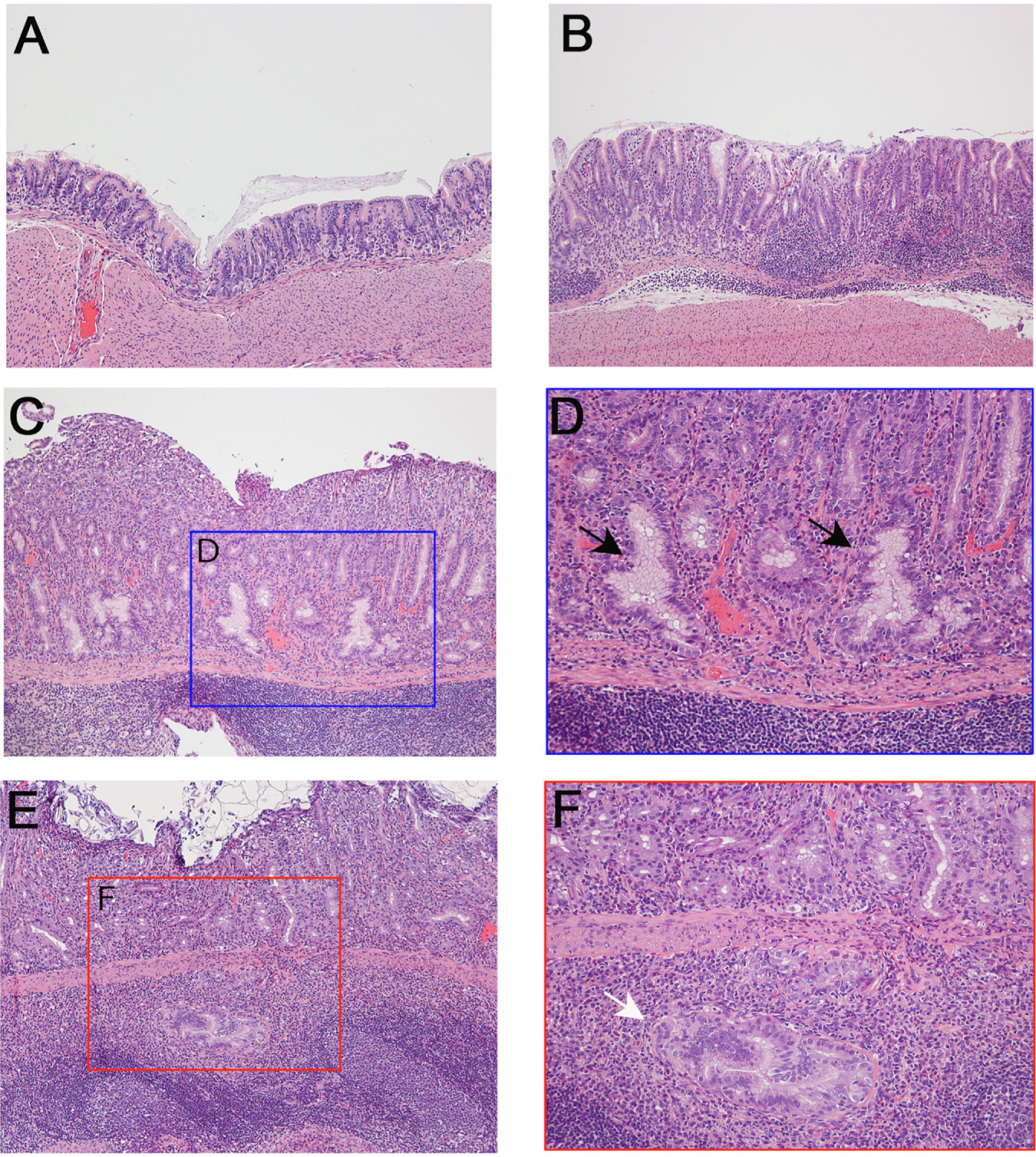
Gastric histology from representative animals. (A) Normal histology from control uninfected animal. (B to F) Gastric tissue from H. pylori-infected animals demonstrating gastric inflammation (B), low-grade dysplasia (C and D), or invasive adenocarcinoma (E and F). Panel D is an enlargement of panel C, showing dysplastic glands by black arrows, and the panel F is an enlargement of panel E, showing invasive adenocarcinoma by white arrow. Magnification, ×100 (A, B, C, and E) and ×200 (D and F).

**FIG 3:**
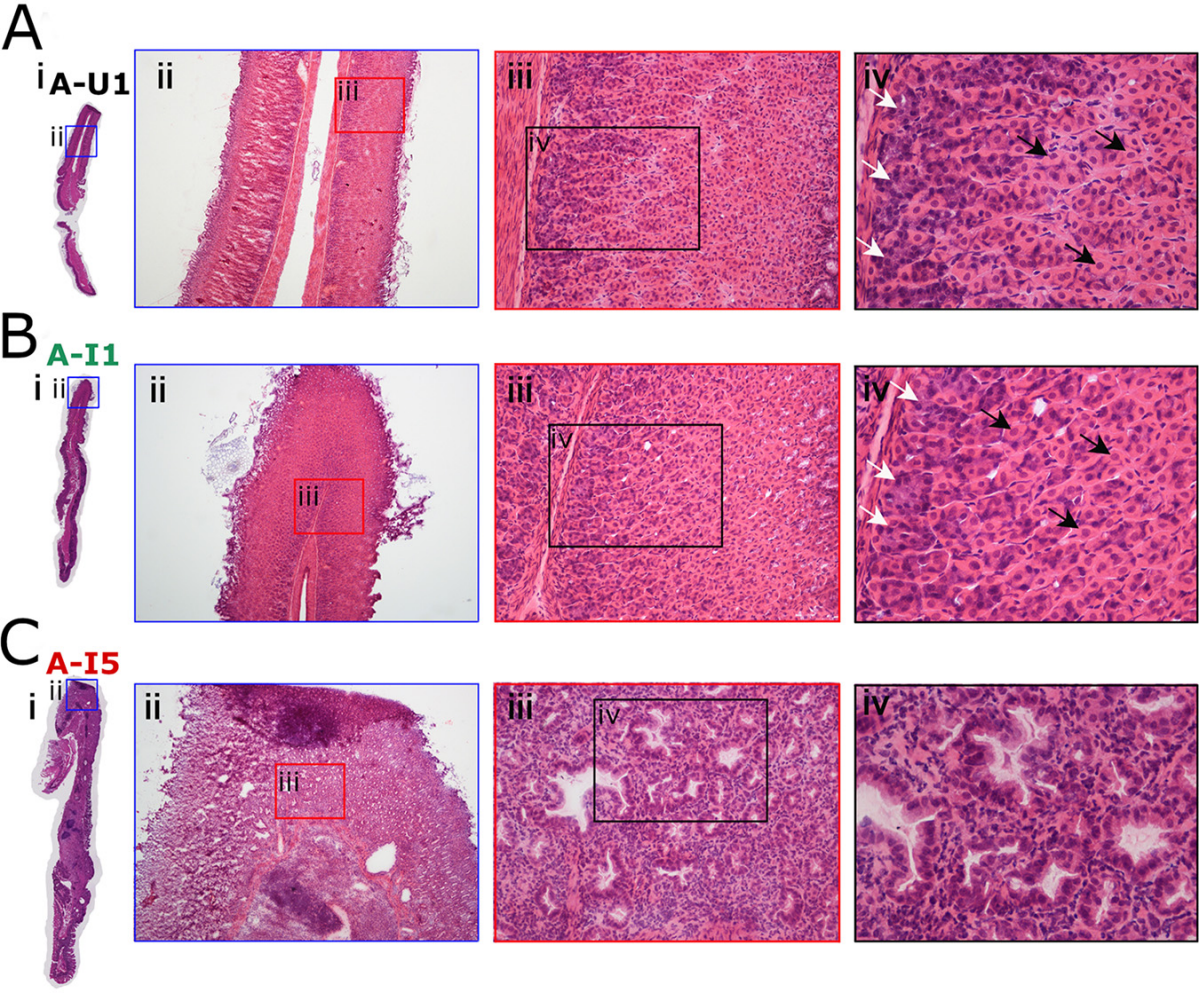
Loss of parietal cells and chief cells in response to H. pylori infection. (A) Normal gastric histology in a representative uninfected animal (A-U1, black font) with intact parietal cells and chief cells in the gastric corpus. (B) Gastric corpus histology in an infected animal with gastritis only (A-I1, green font), showing intact parietal cells and chief cells. (C) Atrophic gastritis in the corpus of infected animal with invasive adenocarcinoma (A-I5, red font), showing loss of parietal and chief cells, replaced by dysplastic glands. White arrows indicate chief cells, and black arrows indicate parietal cells. Magnification, ×40 (ii), ×200 (iii), and ×400 (iv).

When choosing gastric tissues for an initial IMS analysis, we selected stomachs from 4 H. pylori-infected animals that exhibited severe gastric disease (severe gastric inflammation and atrophic gastritis, along with dysplasia or gastric cancer), 1 infected animal with milder gastric disease (mild gastric inflammation without detectable dysplasia or gastric cancer), and 5 control animals (uninfected). The label “A-U” and subsequent numbers (1, 2, 3, 4, and 5) represent designations of individual uninfected animals from gerbil cohort A. The label “A-I” and subsequent numbers (1, 2, 3, 4, and 5) represent designations of individual H. pylori-infected animals from gerbil cohort A, with disease state indicated by color codes in the figures. Gastric inflammation was graded for severity by analyzing the H&E-stained tissues, using the scoring system described in Materials and Methods. Uninfected gerbils exhibited no gastric inflammation. All 5 of the stomachs from H. pylori-infected animals exhibited gastric inflammation and increased numbers of gastric lymphoid follicles compared to uninfected animals (Fig. 1 and 2). Dysplasia was detected in stomachs from two infected animals (Fig. 2C and D), corresponding to A-I3 and A-I4 (colored blue in Fig. 1), and invasive adenocarcinoma was detected in stomachs from two additional infected animals (Fig. 2E and F), corresponding to A-I2 and A-I5 (colored red in Fig. 1). The 4 stomachs from H. pylori*-*infected animals with atrophic gastritis (characterized by inflammation in the gastric corpus and loss of parietal cells and chief cells) exhibited inflammation in both the antrum and the corpus, whereas the stomach from an H. pylori*-*infected animal with mild disease exhibited inflammation only in the antrum (Fig. 1A to D). These 4 stomachs were larger in size than the stomachs from uninfected animals, consistent with hyperplasia. The overall severity of gastric inflammation (combined analysis of antrum and corpus) and number of lymphoid follicles in the four infected animals with severe disease was greater than the severity of inflammation and number of lymphoid follicles in the infected animal with mild disease (AI-1) (Fig. 1 and 3). Uninfected animals exhibited no gastric inflammation or lymphoid follicles.

### Visualization of gastric lipids by imaging mass spectrometry.

**FIG 4:**
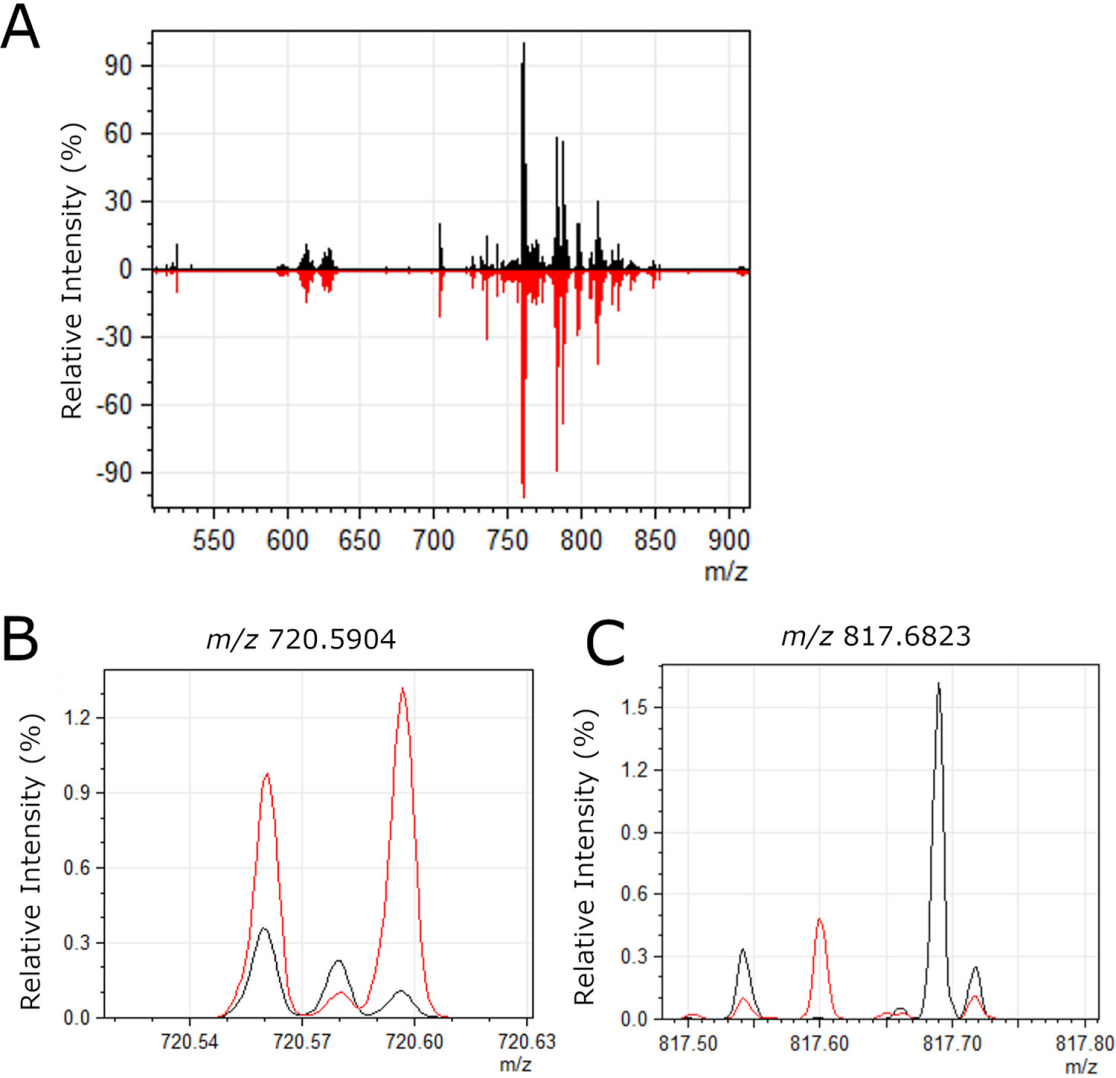
Representative spectra of lipids detected in gastric tissue. (A) Average mass spectra over entire gastric tissue sections are shown for a representative uninfected stomach (black) and a representative H. pylori-infected stomach (red). The overall signal intensity and quality were very similar in infected versus uninfected tissue. (B) Zoomed-in image of the region surrounding *m/z* 720.5904, which was more abundant in infected tissues compared to uninfected, as shown in Fig. 5. (C) Zoomed-in image of the region surrounding *m/z* 817.6823, which was less abundant in infected tissues compared to uninfected, as shown in Fig. 6.

To assess the relative abundance and spatial distribution of lipids in the gastric mucosa, gastric tissues were subjected to IMS analysis. Lipid images were obtained on a 15T Fourier transform ion cyclotron resonance mass spectrometer (FT-ICR MS) (Bruker Daltonics, Billerica, MA) at 75 μm spatial resolution in both positive and negative modes. The lipid images were analyzed as described in Materials and Methods to identify ions that differed in abundance when comparing gastric tissues from infected animals with tissues from uninfected animals. This list of ions was subsequently curated by eliminating likely isotopic signals as well as low-intensity signals, resulting in a list of 36 differentially abundant ions (Table S1). These *m/z* values were searched against the LIPID MAPS database with a mass window of ±0.01 amu (∼13 ppm mass error) to provide tentative identifications based on accurate mass (Table S1) (23). The FT data sets typically resulted in mass accuracies of better than 5 ppm. Some of these ions were increased in abundance in tissues from H. pylori-infected animals compared to tissues from uninfected animals, and others were decreased in abundance in tissues from H. pylori-infected animals compared to tissues from uninfected animals (Table S1). Representative spectra are shown in Fig. 4.

10.1128/mSphere.00826-21.1 TABLE S1 Characteristics of differentially abundant lipids. Download Table S1, XLSX file, 0.01 MB. Copyright © 2021 Lin et al. 2021 Lin et al. https://creativecommons.org/licenses/by/4.0/ This content is distributed under the terms of the Creative Commons Attribution 4.0 International license.

### Identification of lipids by liquid chromatography-mass spectrometry.

**FIG 5:**
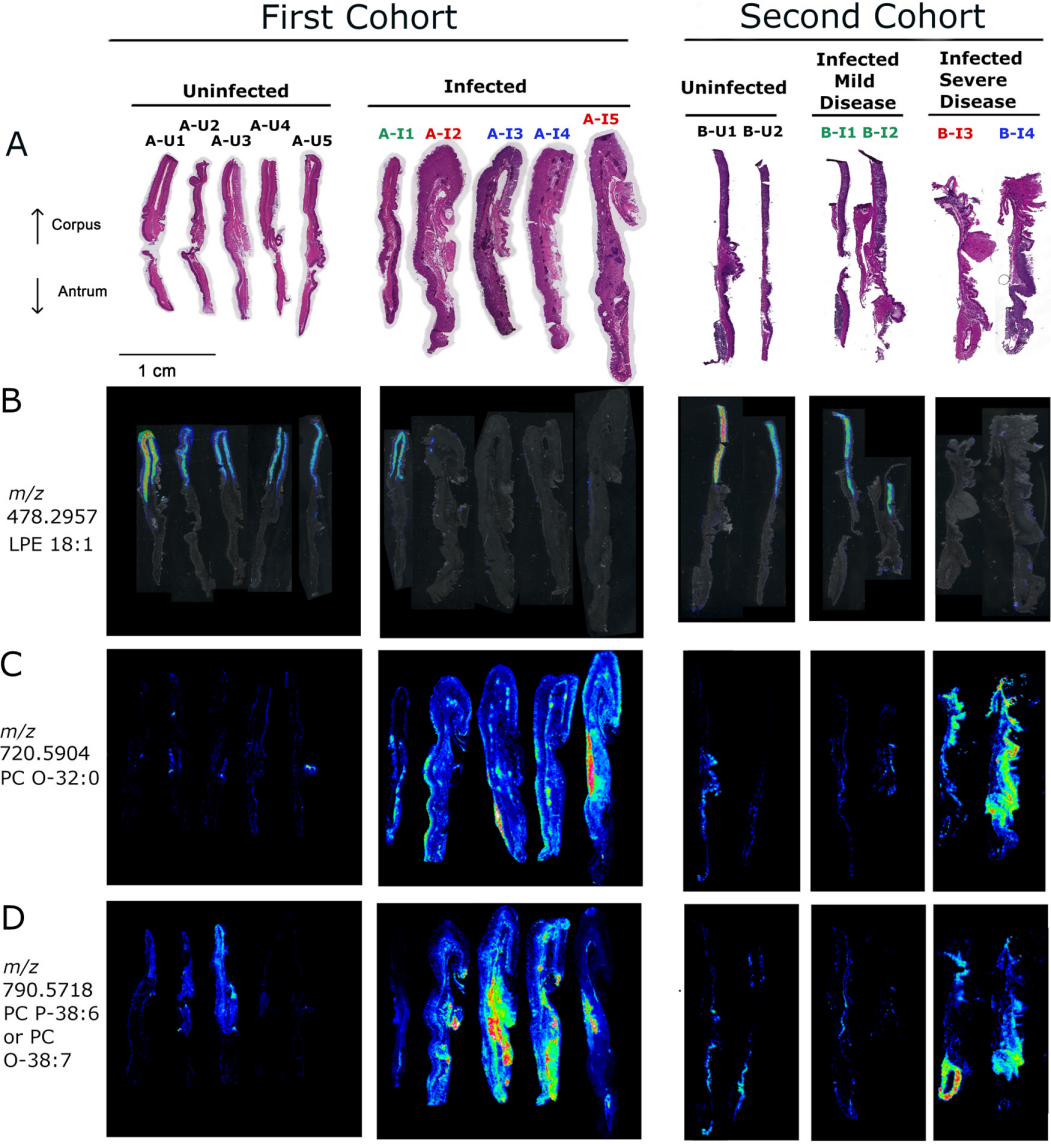
IMS analysis showing differentially abundant lipids detected by LC-MS/MS. (A) H&E staining of the gastric mucosae from uninfected and infected animals in the first cohort (left) and the second cohort (right). (B) Overlay of corpus-specific ion 478.2957 on the optical images of tissue sections for the first (left) and second (right) set of tissues. (C and D) The respective ion images show localization of lipids identified by LC-MS/MS at *m/z* 720.5904 and 790.5718 for the first (left) and the second (right) set of tissues. Black font corresponds to uninfected animals with normal histology, green font indicates infected animals with mild disease (gastritis only), blue font indicates infected animals with dysplasia, and red font indicates infected animals with invasive gastric adenocarcinoma. The figure shows multiple tissue images joined together in an order different from that on the original slides.

To provide a more definitive identification of differentially abundant lipids, several additional sections from one H. pylori-infected animal with gastric adenocarcinoma (A-I5) and one uninfected control stomach (A-U5) were homogenized and subjected to lipid extraction and analysis by LC-MS/MS, as described in Materials and Methods. A total of 11 lipids identified by the LC-MS/MS analysis (Table 1 and Fig. S2 to S6) were correlated (based on accurate *m/z*) with the species detected by IMS. Five ions were increased in abundance, and six were decreased in abundance in stomachs from H. pylori-infected animals compared to stomachs from uninfected controls (Table 1). One of the ions (*m/z* 478.2957, identified as lysophosphatidylethanolamine 18:1 [LPE 18:1]), was localized preferentially to the corpus portion of the stomachs from uninfected animals and the infected animal with mild disease and was decreased in abundance in stomachs from animals with severe disease (Fig. 5). On-tissue fragmentation was undertaken to provide further support for the identification of specific lipids identified by LC-MS/MS. Many ions were not of sufficient intensity to obtain any meaningful fragmentation, but this approach supported the identification of several phosphatidylcholine (PC) species, as described in Materials and Methods.

**TABLE 1:** Lipids observed in positive or negative ion mode IMS correlated with LC-MS/MS results

Lipid class^*e*^	IMS value			LC-MS/MS value		
	ID	FT *m/z*^*a*^	Error^*b*^	ID	LC-MS/MS *m/z*^*c*^	error^*b*^
Sphingomyelin	SM d34:2	701.5595	0.4	SM (18:1/16:1) [M+H]^+^	701.5602	1.7
	SM d36:2	729.5904	0.1	SM (18:2/18:0) [M+H]^+^	729.5901	0.1
	SM d38:2	757.6215	0.4	SM (18:0/20:2) [M+H]^+^	757.6221	0.1
Phosphatidylcholine	PC (P-32:0)	718.5739	0.8	PC (P-32:0) [M+H]^+^	718.5746	0.8
	PC (O-32:0)	720.5904	0.3	PC (O-32:0) [M+H]^+^	720.5903	0.4
	PC (P-38:6)^*d*^	790.5718	3.4	PC (P-38:6) [M+H]^+^	790.5738	0.8
	PC (O-38:6)	792.5885	2.1	PC (O-38:6) [M+H]^+^	792.5872	3.7
	PC 34:4	754.5364	2.2	PC (34:4) [M+H]^+^	754.5372	1.1
Phosphatidylethanolamine	PE 36:2	742.5402	1.3	PE (18:1:18:1) [M−H]/PE (18:0/18:2) [M−H]^−^	742.5378	1.8
Lysophosphatidylethanolamine	LPE 18:1	478.2957	3.8	LPE 18:1 [M−H]^−^	478.2923	3.3
Lysophosphatidyl-inositol	LPI 16:0	571.2891	0.3	LPI (16:0) [M−H]^−^	571.2872	2.9

a Fourier transform ion cyclotron resonance mass spectrometry mass-to-charge ratio.

b Mass error (ppm), based on comparison to the calculated theoretical value.

c Liquid chromatography-mass spectrometry mass-to-charge ratio.

d Possible isomers (ions with identical chemical formulas; see Table S1 in the supplemental material).

e SM, sphingomyelin; PC, phosphatidylcholine; PE, phosphatidylethanolamine; LPE, lysophosphatidylethanolamine; LPI, lysophosphatidyl-inositol.

10.1128/mSphere.00826-21.3 FIG S2 ESI mass spectra (left) and MS/MS spectra (right) for sphingomyelins. (A) SM (34:2) in positive ionization mode at [M+H]^+^ 701.5602. (B) SM (36:2) in positive ionization mode at [M+H]^+^ 729.5901. (C) SM (38:2) in positive ionization at [M+H]^+^ 757.6221. Theoretical MH^+^ *m*/*z* values are listed under the structures. Download FIG S2, TIF file, 1.1 MB. Copyright © 2021 Lin et al. 2021 Lin et al. https://creativecommons.org/licenses/by/4.0/ This content is distributed under the terms of the Creative Commons Attribution 4.0 International license.

10.1128/mSphere.00826-21.4 FIG S3 ESI mass spectrum (left) and MS/MS spectrum (right) for phosphatidylcholines. (A) PC(P-32:0)/PC(O-32:1)/PC(31:1) in positive ionization mode at [M+H]^+^ 718.5746. Note, PC(P-32:0) is shown as one example. (B) PC(O-32:0)/PC(31:0) in positive ionization mode at [M+H]^+^ 720.5903. Note PC(O-32:0) is shown as one example. (C) PC(P-38:6)/PC(37:7)/PC(O-37:0)/PC(36:0) in positive ionization mode at [M+H]^+^ 790.5738. Note, PC(P-38:6) is shown as one example. (D) PC(O-38:6)/PC(P-38:5)/PC(37:6) in positive ionization mode at [M+H]^+^ 792.5872. Note, PC(O-38:6) is shown as one example. (E) PC (34:4) in positive ionization mode at [M+H]^+^ 754.5372. Theoretical MH^+^ *m*/*z* values are listed under the structures. Download FIG S3, TIF file, 1.6 MB. Copyright © 2021 Lin et al. 2021 Lin et al. https://creativecommons.org/licenses/by/4.0/ This content is distributed under the terms of the Creative Commons Attribution 4.0 International license.

10.1128/mSphere.00826-21.5 FIG S4 ESI mass spectrum (left) and MS/MS spectrum (right) for PE (18:0/18:2) in negative ionization mode at [M−H]^−^ 742.5378. Theoretical [M−H]^−^ *m*/*z* values listed under the structure. Download FIG S4, TIF file, 0.3 MB. Copyright © 2021 Lin et al. 2021 Lin et al. https://creativecommons.org/licenses/by/4.0/ This content is distributed under the terms of the Creative Commons Attribution 4.0 International license.

10.1128/mSphere.00826-21.6 FIG S5 ESI mass spectrum (left) and MS/MS spectrum (right) for Lyso PE (18:1) in negative ionization mode at [M−H]^−^ 478.2923. Theoretical [M−H]^−^ *m*/*z* values listed under the structure. Download FIG S5, TIF file, 0.3 MB. Copyright © 2021 Lin et al. 2021 Lin et al. https://creativecommons.org/licenses/by/4.0/ This content is distributed under the terms of the Creative Commons Attribution 4.0 International license.

10.1128/mSphere.00826-21.7 FIG S6 ESI mass spectrum (left) and MS/MS spectrum (right) for Lyso PI (16:0) in negative ionization mode at [M−H]^−^ 571.2872. Theoretical [M−H]^−^ *m*/*z* values listed under the structure. Download FIG S6, TIF file, 0.3 MB. Copyright © 2021 Lin et al. 2021 Lin et al. https://creativecommons.org/licenses/by/4.0/ This content is distributed under the terms of the Creative Commons Attribution 4.0 International license.

### Differentially abundant lipids detected in tissues from an independent cohort of animals.

**FIG 6:**
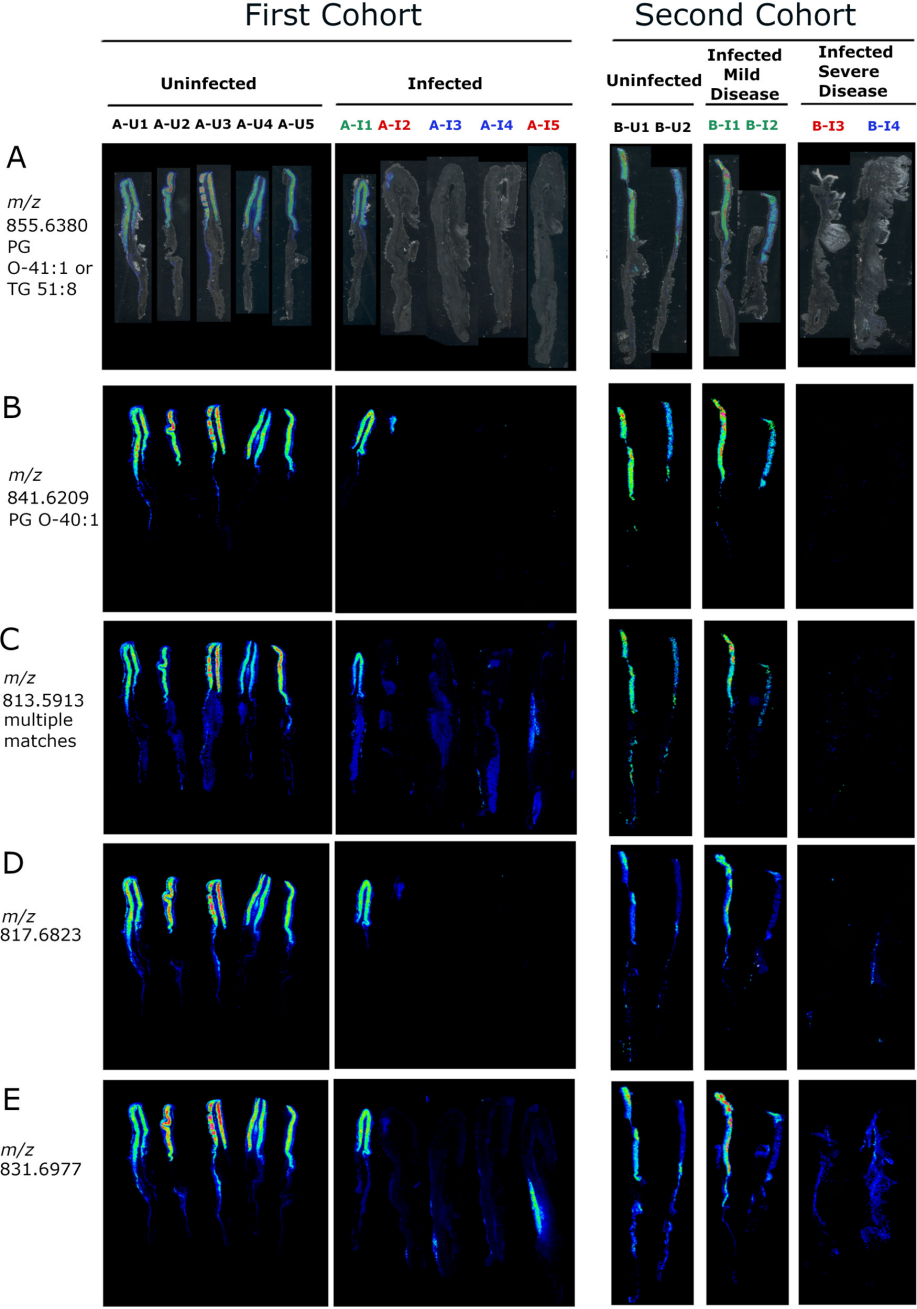
IMS analysis showing corpus-specific localization of positive ions decreased in atrophic gastritis. (A) Overlay of ion 855.6380 on tissue optical sections for the first (left) and second (right) cohort of animals. (B to E) The respective ion images show corpus-specific localization of ions *m/z* 841.6209, 813.5913, 817.6823, and 831.6977 in the first (left) and second (right) set of tissues. Black font corresponds to uninfected animals with normal histology, green font indicates infected animals with mild disease (gastritis only), blue font indicates infected animals with dysplasia, and red font indicates infected animals with invasive gastric adenocarcinoma. The figure shows multiple tissue images joined together in an order different from that on the original slides.

**FIG 7:**
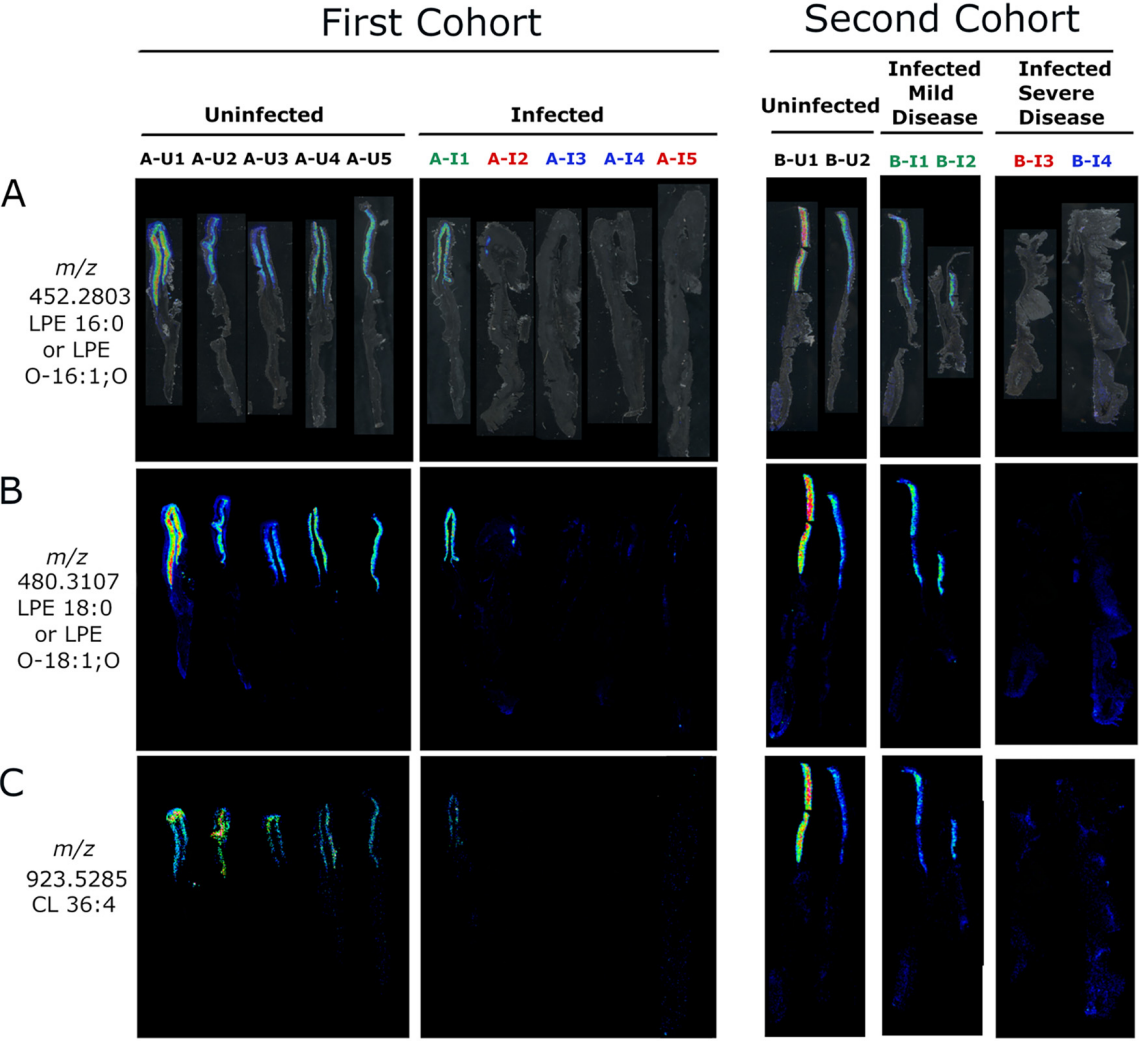
IMS analysis showing corpus-specific localization of negative ions decreased in atrophic gastritis. (A) Overlay of ion 452.2803 on tissue optical sections for the first (left) and second (right) cohort of animals. (B and C) The respective ion images show corpus-specific localization of ions *m/z* 480.3107 and 923.5285 in the first (left) and second (right) set of tissues. Black font corresponds to uninfected animals with normal histology, green font indicates infected animals with mild disease (gastritis only), blue font indicates infected animals with dysplasia, and red font indicates infected animals with invasive gastric adenocarcinoma. The figure shows multiple tissue images joined together in an order different from that on the original slides.

To evaluate the reproducibility of the observed lipid alterations and further assess a possible relationship between gastric disease severity and lipid alterations, we analyzed archived frozen gastric tissues from a separate cohort of gerbils (cohort B; 4 infected and 2 uninfected animals). None of the uninfected stomachs exhibited gastric inflammation, but all four of the stomachs from H. pylori*-*infected animals in this cohort had gastric inflammation (Fig. S7). Two of the infected animals selected for analysis (labeled B-I3 and B-I4 in Fig. 5 and 7) had severe gastric disease (dysplasia or invasive carcinoma). The gastric tissues from these two animals exhibited atrophic gastritis (loss of parietal and chief cells in the corpus) and overall inflammation scores of 12 and 11.5 on a 12-point scale (Table 2 and Fig. S7). Overall, the tissues from these two animals exhibited features similar to those of tissues from the 4 animals with severe gastric disease in the first cohort, including atrophic gastritis, thickening of tissue consistent with hyperplasia, and inflammation in both the antrum and the corpus. The other two infected animals (labeled B-I1 and B-I2 in Fig. 4 and 6) had mild gastritis, with no loss of parietal or chief cells (inflammation scores of 0.5 and 2.5, respectively). The tissues from B-I1 and BI-2 exhibited inflammation only in the antrum and had histologic features similar to those observed in tissue from the single animal with mild disease (A-I1) in the first cohort. Among the 11 differentially abundant lipids detected by LC-MS/MS in tissues from the first cohort of gerbils, 5 were confirmed to be differentially abundant based on IMS analysis of tissues from the second cohort of gerbils (Fig. 5 and Table S1). Similarly, 20 other differentially abundant lipids tentatively identified by accurate mass but not detected by LC-MS/MS were differentially abundant in both the first and second cohorts of tissue (Table S1), exemplified by the lipids shown in Fig. 6 and 7. Further support for the tentative identifications of differentially abundant lipids was provided by experiments using ammonium formate rinses, which typically reduce sodium and potassium adducts, as described in Materials and Methods and Table S1.

**TABLE 2:** Quantification of parietal cell loss and chief cell loss

Gerbil^*a*^	% parietal cell loss	% chief cell loss	Oxyntic gland atrophy score^*b*^
A-U1	0	0	0
A-U2	0	0	0
A-U3	0	0	0
A-U4	0	0	0
A-U5	0	0	0
B-U1	0	0	0
B-U2	0	0	0
A-I1	0	0	0
A-I2	60	75	2
A-I3	100	100	3
A-I4	100	100	3
A-I5	95	100	3
B-I1	0	0	0
B-I2	0	0	0
B-I3	>75	100	3
B-I4	>75	100	3

a Uninfected gerbils are designated with a “U” in the name, and H. pylori-infected gerbils are designated with an “I” in the name. Animals from the first cohort are designated with names beginning with “A,” and animals from the second cohort are designated with names beginning with B.”

b Oxyntic gland atrophy is a 0 to 3 score based on the combination of parietal and chief cell loss as follows: 0, no atrophy; 0.5, <25% parietal and <25% chief cell loss; 1.0, >25% parietal cell loss but <50% chief cell loss; 1.5, 50% parietal cell loss and 50% chief cell loss; 2.0, 75% parietal cell loss and 75% chief cell loss; 2.5, 75% parietal cell loss and 90% chief cell loss; 3.0, >75% parietal cell loss and 100% chief cell loss.

10.1128/mSphere.00826-21.8 FIG S7 Inflammation in the second cohort of animals. (A and B) Acute and chronic inflammation in corpus. (C and D) Acute and chronic inflammation in antrum. (E) Total inflammation scores (combined analysis of corpus and antrum). (F) Number of lymphoid follicles visible in gastric mucosa. Gastric inflammation was scored as described in Materials and Methods. The label “U” and subsequent numbers (1 and 2) represent designations of individual uninfected animals. The label “I” and subsequent numbers (1, 2, 3, and 4) represent designations of individual H. pylori-infected animals, with disease diagnosis indicated by color codes. Uninfected animals are represented by black circles, infected animals with mild disease (gastritis only) by green squares, infected animals with dysplasia by blue circles, and infected animals with invasive gastric adenocarcinoma by red diamonds. Download FIG S7, TIF file, 0.4 MB. Copyright © 2021 Lin et al. 2021 Lin et al. https://creativecommons.org/licenses/by/4.0/ This content is distributed under the terms of the Creative Commons Attribution 4.0 International license.

### Spatial distribution of differentially abundant lipids.

We then undertook further analysis of the tissues from both the first and second cohorts of animals to assess the spatial distribution of the differentially abundant lipids identified by IMS and compare the ion images with the respective histologic images. Most of the lipids that were increased in abundance in tissues from H. pylori-infected animals compared to tissues from uninfected animals were localized in both antrum and corpus regions of the stomach (Fig. 5 and Table S1). The increased abundance of these lipids was most evident when comparing tissues from H. pylori-infected animals with severe gastric disease with tissues from uninfected animals (Fig. 5C and D). Differences were less prominent when comparing tissues from infected animals with mild disease to tissues from uninfected animals.

Sixteen of the 36 differentially abundant lipids were localized preferentially in the corpus region of the stomach (Fig. 6 and 7; Table S1). These lipids were visualized in the gastric corpus from uninfected animals and infected animals with mild disease but were decreased in abundance in tissues from H. pylori-infected animals with severe gastric disease. Among these 16 corpus-specific lipids, 13 were detected in both the first and second cohort by IMS. One of these, LPE 18:1, was detected by both IMS and LC-MS/MS (Fig. 5A). The tissue specimens from H. pylori-infected animals with milder disease either resembled the uninfected tissue specimens or had features intermediate between those of uninfected tissues and those of infected animals with severe disease (Fig. 5A; Fig. 6 and 7). Probable identifications for these ions include phosphatidylglycerols, triglycerides, lysophosphatidylethanolamines, and cardiolipins (Table S1). In summary, these experiments identified numerous lipids that were detectable and localized mainly to the gastric corpus in uninfected animals and H. pylori*-*infected animals with mild disease, but essentially absent in H. pylori*-*infected animals with severe disease.

## DISCUSSION

In this study, we utilized IMS as an unbiased approach to evaluate gastric lipid alterations that occur in response to H. pylori infection. We observed that specific gastric lipids were increased in abundance in H. pylori-infected animals compared to uninfected animals, and others were decreased in abundance in response to H. pylori infection or H. pylori*-*induced disease. IMS also allowed us to define the spatial distributions of individual lipids of interest within the stomach. Some of the differentially abundant lipids were localized specifically to the corpus, whereas others were localized throughout the stomach. The use of LC-MS/MS provided additional supportive evidence for the molecular identity of several of the differentially abundant lipids.

To evaluate the reproducibility of the initial results (first animal cohort), we analyzed archived frozen gastric tissues from a different study (second animal cohort). In both cases, gastric tissues from H. pylori-infected animals were compared with gastric tissues from uninfected animals. Twenty-five lipids were differentially abundant (comparing *H. pylori*-infected and uninfected tissues) in both the first animal cohort and the second animal cohort (Fig. 5 to 7 and Table S1 in the supplemental material). An additional 11 lipids were differentially abundant in the first animal cohort but not in the second animal cohort (Table S1). The lack of perfect agreement between the two experiments might be attributable to differences in the two cohorts of Mongolian gerbils (which are outbred) or differences in the diets administered to the two cohorts of gerbils (described in Materials and Methods).

Increased abundance of lipids in H. pylori-infected stomachs could potentially reflect increased lipid production or decreased lipid turnover within gastric tissue, alterations in the membrane composition of various cells in gastric tissue, or infiltration of inflammatory cells producing these lipids. IMS has been used successfully to detect lipids and other products of bacterial origin (24–26). In the current study, it is unlikely that the signals detected correspond to H. pylori lipids, and they more likely correspond to lipids of gerbil origin. First, any lipids that are present in uninfected animals but reduced in abundance in H. pylori-infected animals are not derived from H. pylori. Furthermore, the distribution of many of the lipids in regions of gastric tissue deeper than the mucosal layer is more consistent with host-associated lipids than lipids associated with the bacteria since H. pylori typically localizes in either the superficial gastric mucus layer or gastric glands without invading host tissue (27). Moreover, H. pylori was not detected by Steiner staining in stomachs from several of the experimentally infected animals with severe disease. Lipids with decreased abundance in H. pylori-infected stomachs could potentially reflect loss of specific gastric cell types in the setting of H. pylori infection, decreased lipid production or increased utilization within gastric tissue, or alterations in the membrane composition of various cells in gastric tissue.

Many of the differentially abundant lipids were detected specifically in the gastric corpus from uninfected animals and were less abundant in corresponding tissue from infected animals exhibiting atrophic gastritis (characterized by a loss of gastric parietal and chief cells). The observed differences in lipid abundance might reflect changes in these cellular populations in the gastric corpus. The IMS methodology used in this study did not have sufficiently high spatial resolution to permit assignment of the lipids to specific host cell types, but we speculate that the lipids localized to the gastric corpus might be constituents of specialized cell types such as parietal cells or chief cells, which are present in the gastric corpus but not the gastric antrum. Indeed, we observed loss of parietal and chief cells in the tissues from animals with severe gastric disease in two different animal cohorts. Parietal cells are characterized by a complex network of intracellular membranes (28), which might be a site for localization of distinctive lipids.

Thus far, there has been relatively little effort to systematically examine alterations in gastric lipids that accompany H. pylori infection or H. pylori-related disease states (such as atrophic gastritis or gastric cancer) in humans. Several studies reported that H. pylori infection leads to reduced concentrations of phospholipids (including PC and phosphatidylethanolamine [PE]) in the human stomach (29–33), whereas another study reported increased levels of PE in H. pylori*-*positive individuals compared to H. pylori-negative individuals (34). Although the localization of specific lipids in human gastric tissue has not been examined in detail, one recent study reported the detection of three highly expressed lipid molecules (sphingomyelin [SM] and PCs) in human gastric mucosa using IMS (35).

H. pylori-induced alterations in gastric lipids are likely to have numerous functional consequences. Phospholipids (PE, phosphatidylglycerol [PG], and PC) act as gastric surfactants, forming a protective hydrophobic layer on the gastric luminal surface (36). Disruption of this barrier potentially increases the susceptibility of gastric mucosal cells to damage. Furthermore, lipids have a pivotal role in many cellular processes, including cell signaling, so alterations in cellular lipid composition could potentially lead to disruptions in cellular processes that are relevant for carcinogenesis (37, 38). Consistent with this hypothesis, previous studies have reported altered abundance of lipids in sites of gastric cancer compared to adjacent nonneoplastic mucosa (39). Gastric lipid alterations are also predicted to influence interactions between H. pylori and host cells. For example, previous studies have shown that H. pylori can bind to PE and LPE on gastric cells (40–42) and that SM acts as a receptor for the secreted H. pylori VacA toxin (43). Therefore, alterations in these gastric lipids might influence H. pylori adherence to host cells or the extent of VacA-induced cellular alterations.

In summary, this study shows that there are changes in the abundance of specific lipids in gastric tissue in response to H. pylori infection and H. pylori*-*mediated disease. These alterations are predicted to have important functional consequences relevant to H. pylori-host interactions and the pathogenesis of gastric cancer. The observed loss of multiple lipids from the gastric corpus in H. pylori-infected animals with atrophic gastritis illustrates that this condition is associated with extensive molecular alterations in the gastric corpus. The mechanisms by which H. pylori infection can lead to atrophic gastritis are incompletely understood, and relatively few molecular markers for this condition have been described (44). Detecting a loss of specific lipids in the setting of atrophic gastritis potentially provides a new approach for detecting this premalignant condition.

## MATERIALS AND METHODS

### Infection of Mongolian gerbils with H. pylori.

Male Mongolian gerbils (35 to 45 g weight) were obtained from Charles River Laboratories. Gerbils were fasted overnight and then were infected via oral gavage (day 0 and day 2) with a 0.5-ml suspension of H. pylori strain 7.13 (1 × 10^9^ CFU/ml) in Brucella broth (13, 16, 17, 19). Gerbils in the first cohort were fed an AIN-93M rodent diet (Bio-Serv), beginning 7 days prior to infection and throughout the remainder of the experiment. In parallel, uninfected gerbils received the same diet. The second cohort of gerbils was infected using the same methods but were fed an AIN-93M diet with high salt content (to increase disease severity) and supplemental iron (17). Specifically, gerbils in the second cohort were fed an AIN-93M rodent diet containing 8% (wt/wt) sodium chloride and supplemental iron (250 mg/kg) (Bio-Serv) beginning 7 days prior to infection and throughout the remainder of the experiment. In parallel, uninfected gerbils received the same diet. Uninfected control animals in the second cohort received Brucella broth by gavage at the beginning of the experiment, whereas uninfected control animals in the first cohort did not undergo gastric manipulation. Animals were euthanized at 3 months postinfection.

### Processing of gastric tissue.

Gerbil stomachs were processed to retain the glandular portion of the stomach (corpus and antrum), and the nonglandular portion (forestomach) was discarded (13, 17, 19). The glandular portion was cut open along the lesser curvature and laid flat (Fig. S1 in the supplemental material). Half of the glandular section of stomach was flash-frozen in a plastic cassette with dry ice and then stored at −70°C until it was used for subsequent IMS analysis, and another portion was used for FFPE histology analysis as described below (Fig. S1).

### Histology.

Longitudinal strips of stomach tissues were fixed in 10% neutral buffered formalin overnight, embedded in paraffin, sectioned, and stained with hematoxylin and eosin. Histologic sections of gastric tissues were analyzed by a gastrointestinal pathologist in a blinded fashion. Inflammation scores (0, 1, 2, and 3, corresponding to absent, mild, moderate, or marked inflammation, respectively) were assigned to evaluate both acute (neutrophils) and chronic (mononuclear leukocytes) inflammation. The inflammation was evaluated in both the corpus and antrum, and these scores were added together to yield a cumulative score of 0 to 12 (13, 17–19, 45). The histologic evaluation also included an evaluation of whether gastric ulceration, dysplasia, or gastric adenocarcinoma were present (45). The abundance of parietal cells and chief cells in the gastric corpus was scored, using a previously described method (13). H. pylori was detected by Steiner staining in 1 of 5 experimentally animals in the first cohort (AI-3) and 3 of 4 animals experimentally infected in the second cohort (BI-1, BI-3, and BI-4) at the endpoint of the experiment. Loss of H. pylori in the stomach is not unexpected in the setting of atrophic gastritis and has been previously reported in humans (46).

### Imaging mass spectrometry.

The fresh-frozen glandular tissues were affixed to a cryostat chuck (Leica CM 3050S) with optimal cutting temperature polymer (OCT) used as a “glue” to facilitate generation of thin sections (12 μm). The sections were thaw-mounted onto indium-tin oxide (ITO)-coated glass slides (Delta Technologies, Ltd., Loveland, CO). Serial sections were obtained for lipid imaging (positive and negative ion mode) and H&E staining. Tissues were placed in a slide mailer, flushed with nitrogen gas, sealed, and stored at −80°C until analysis. Prior to analysis, the samples were removed from the freezer and allowed to warm up to room temperature while still sealed in a benchtop desiccator. One set of tissue sections was subjected to a rinse with ammonium formate (50 mM cold ammonium formate, three rinses for 5 s each) to remove salts prior to matrix application (47). 1,5-Diaminonaphthalene (Sigma-Aldrich) was used as the matrix for MALDI imaging and was applied to the tissues by sublimation (48). Briefly, approximately 100 mg of matrix was applied to the bottom of a custom sublimation apparatus and was allowed to sublimate onto the tissues at 130°C and ∼25 mTorr for 4 min (49). Lipid images were acquired on a Solarix 15T FT-ICR MS (Bruker Daltonics, Billerica, MA), with serial sections being analyzed in positive and negative mode at 75 μm spatial resolution. In all cases, uninfected and infected tissues were sectioned, prepared, and analyzed together, although not all samples could fit on a single slide. Each slide contained both uninfected and infected tissues. The instrument is equipped with a Smartbeam II Nd:YAG laser run at 2 kHz and 355 nm. Five hundred shots were acquired per spot, with the stage moving in a random smart walk at each pixel. Data were acquired over the mass range from 345 to 2,000 *m/z* (Fig. 4). Each run was externally calibrated using red phosphorous in both positive and negative ion modes. After data acquisition, data were recalibrated internally using known abundant lipid species, resulting in mass errors of generally <5 ppm.

We used SCiLS (SCiLS Lab 2017a, version 5.00.9510) for analysis of tissues from the initial experiment to find discriminatory peaks between infected and uninfected tissues using receiver operating characteristic (ROC). For the comparisons, no normalization was used. Areas were selected encompassing either the entire stomach or just the corpus or antrum of each tissue specimen. The analysis was performed using various thresholds for the ROC curve (from 0.3 to 0.7) and was performed on the entire data set (not peak picked). The results were in the form of *m/z* intervals that exceeded the thresholds. These data were manually inspected to identify robust signals that differed in abundance when comparing gastric tissues from infected animals with tissues from uninfected animals. We detected 72 ions in positive ion mode and 15 ions in negative ion mode that differed in abundance. This list of ions was subsequently curated by eliminating likely isotopic signals as well as low-intensity signals. We did not use any quantitative criteria for eliminating low-intensity signals. This process resulted in a list of 36 ions (Table S1). flexImaging software (Bruker) was used for visualization of the ion images in tissues from both the first and second animal cohorts, and mMass software was used to visualize averaged mass spectra exported from flexImaging (50). The grouping of uninfected and infected tissues shown in the figures is not identical to the organization on the original slides. Overall intensities of images (showing tissues from both infected and uninfected animals) were optimized individually when necessary.

### Chemicals.

HPLC-grade chloroform, methanol, isopropanol, acetonitrile, and water were purchased from Fisher Scientific (Pittsburg, PA). Formic acid and ammonium formate were obtained from Sigma-Aldrich (St. Louis, MO). *tert*-Butyl methyl ether (MTBE) was purchased from Honeywell (Charlotte, NC). SPLASH Lipidomix mass spectrometry standard was purchased from Avanti Polar Lipids (Alabaster, AL, USA).

### Lipid extraction for liquid chromatography-mass spectrometry procedure.

Three 12-μm sections of fresh-frozen tissue were used for lipid extraction. The corpus and antrum tissues were carefully scraped using a razor and placed in a 1.5-ml HPLC glass vial. One milliliter of MMC extraction mixture (51) (1.3:1:1, methanol/*tert*-butyl methyl ether [MTBE]/chloroform) spiked with 10 μl of lipid standard mixture was added to the same vial, and the preparations were centrifuged at 3,000 rpm for 10 min. The supernatant was transferred to a separate HPLC vial and was evaporated to dryness under nitrogen. The sample was then reconstituted with 30 μl methanol, and 10 μl was subjected to LC-MS/MS analysis.

Chromatographic separation (52) was achieved using an Acquity ultraperformance liquid chromatography (UPLC) BEH C_18_ column (2.1 by 150 mm, 1.7 μm particle size) (Waters Corporation, Milford, MA, USA) held at 50°C coupled to a Vanquish binary pump (Thermo Fisher Scientific, San Jose, CA). A gradient mobile phase was maintained for 55 min with a flow rate of 250 μl/min and was comprised of mobile phase A, 10 mM ammonium formate in 40:60 (vol/vol) water/acetonitrile with 0.1% formic acid, and mobile phase B, 10 mM ammonium formate in 90:10 (vol/vol) isopropanol/acetonitrile with 0.1% formic acid. The gradient elution profile was as follows: 32% B (0 to 1.5 min), 32 to 45% B (1.5 to 4 min), 45 to 52% B (4 to 6 min), 52 to 58% B (6 to 10 min), 58 to 66% B (10 to 16 min), 66 to 70% B (16 to 22 min), 70 to 75% B (22 to 30 min), 75 to 97% B (30 to 38 min), 97% B (38 to 48 min), 97 to 32% B (48 to 50 min), and 32% B (50 to 55 min). Ten microliters of sample were injected via the Vanquish autosampler maintained at 4°C and ionized by a HESI (heated ESI source) and analyzed using Q-Executive HF instrument (Thermo Scientific, San Jose, CA, USA). Data were acquired at both full (MS1) and data-dependent MS2 (ddMS2) scan modes using positive and negative polarities. The full scan mode had a mass resolution of 60,000, mass range of *m/z* 400 to 1,200 in positive polarity and *m/z* 240 to 1,600 in negative polarity, and a maximum trap fill time of 100 ms. ddMS2 data were acquired at 15,000 resolution with a maximum trap fill time of 160 ms. The isolation window of selected MS1 ions was ± 1.4 *m/z* with a normalized collision energy (NCE) of 20% and 25%. LC-MS/MS data were acquired using Xcalibur version 4.0.

### Data processing for LC-MS/MS.

Raw data files (Thermo raw files) consisting of blanks and samples were converted to mgf files using the MSConvert function of ProteoWizard. Mgf and mzXML input files were searched using the spectrum searcher feature of LipiDex software v1.0.2 (53) for lipid identifications. Precursor *m/z*, fragmentation spectra, chromatographic retention time, and identification were integrated using LipiDex. The fragmentation patterns of individual lipid standards were manually interpreted and then matched against LipiDex (53) library results.

### Lipid identification.

Several approaches were utilized to identify the differential ion signals obtained by IMS on the tissue sections. First, the experimental masses determined from the FT-ICR imaging experiments were searched against the LIPID MAPS database (23) with a mass tolerance of 0.01 *m/z* (∼13 ppm). For positive ion mode, protonated ions, sodium adducts, and potassium adducts were searched, while only deprotonated ions were considered for negative ion mode. After internal calibration, experimental masses from the imaging experiments should be accurate to ∼5 ppm, so this wide window was used to ensure any possible matches would not be missed. No more than six possible matches were returned for any individual ion. These data were then cross-referenced with the LC-MS/MS data. In cases where there was no supporting fragmentation evidence (i.e., for many structures containing an odd-chain lipid) or no evidence of the parent ion in any form, these were determined to be unlikely matches. In some cases, that left only one possible species, while in others, exact structural isomers were possible.

MS/MS fragmentation of the parent ions found in MALDI IMS was attempted on tissue on the FT-ICR MS. Many ions were not of sufficient intensity to obtain any meaningful fragmentation. The remaining ions at *m/z* 718.5739, *m/z* 720.5904, and 792.5885 all gave a fragment ion at *m/z* 184, which is consistent with the phosphocholine moiety and supports their identities as PC species. Finally, one set of the second cohort of tissues was analyzed in positive ion mode after ammonium formate rinsing (with all other sample preparation the same). This typically reduces sodium and potassium adducts. None of the putative protonated ions showed a difference in this analysis, while several species tentatively identified as sodium or potassium adducts (i.e., TG 48:8, PE 38:4, PS 38:5, and PE 40:1) were not visible after the ammonium formate rinse, which is consistent with salt adducts of these species. These analyses are summarized in Table S1.

### Ethics statement.

All animal experiments were approved by the Vanderbilt University Institutional Animal Care and Use Committee (protocol M1700055-00).
