# Supplementary figures and images for: Loss of Corpus-Specific Lipids in Helicobacter pylori-Induced Atrophic Gastritis
Source: mSphere. 2021 Nov 24;6(6):e00826-21. doi: 10.1128/mSphere.00826-21 (PMC8612251; doi:10.1128/mSphere.00826-21)

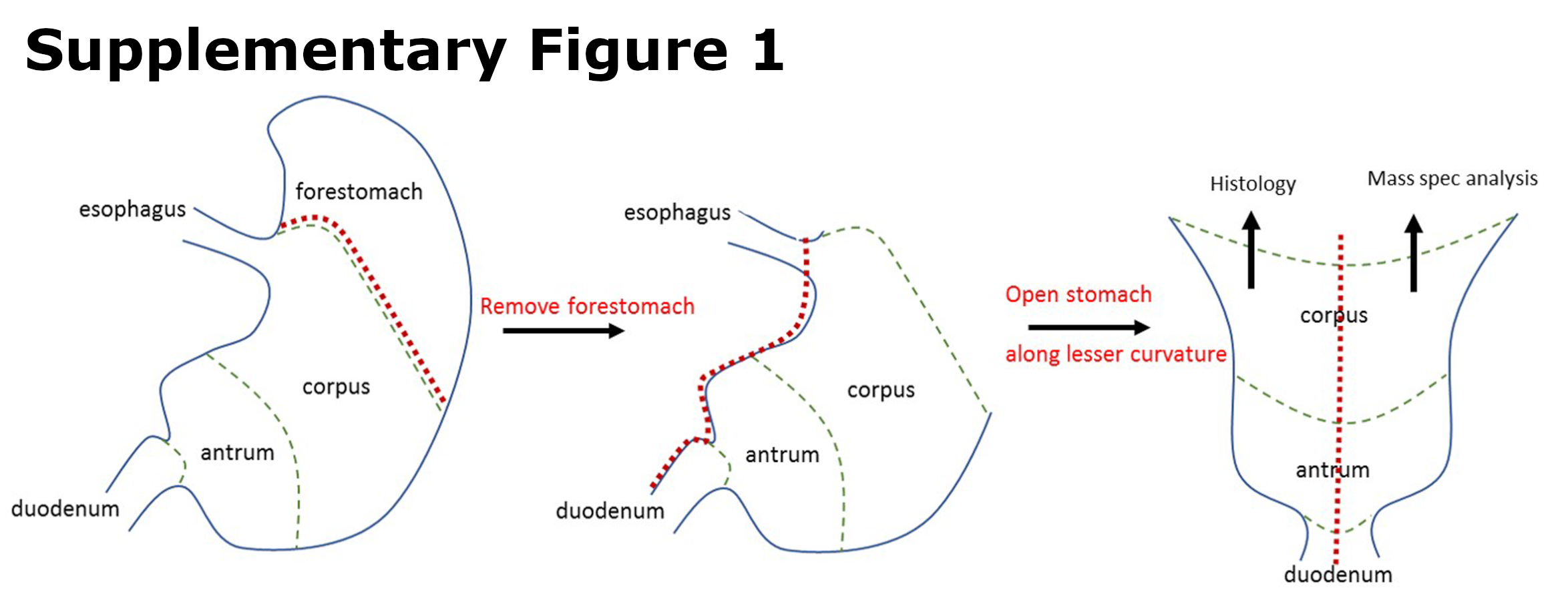

Supplement: FIG S1 [file msphere.00826-21-sf001.tif]

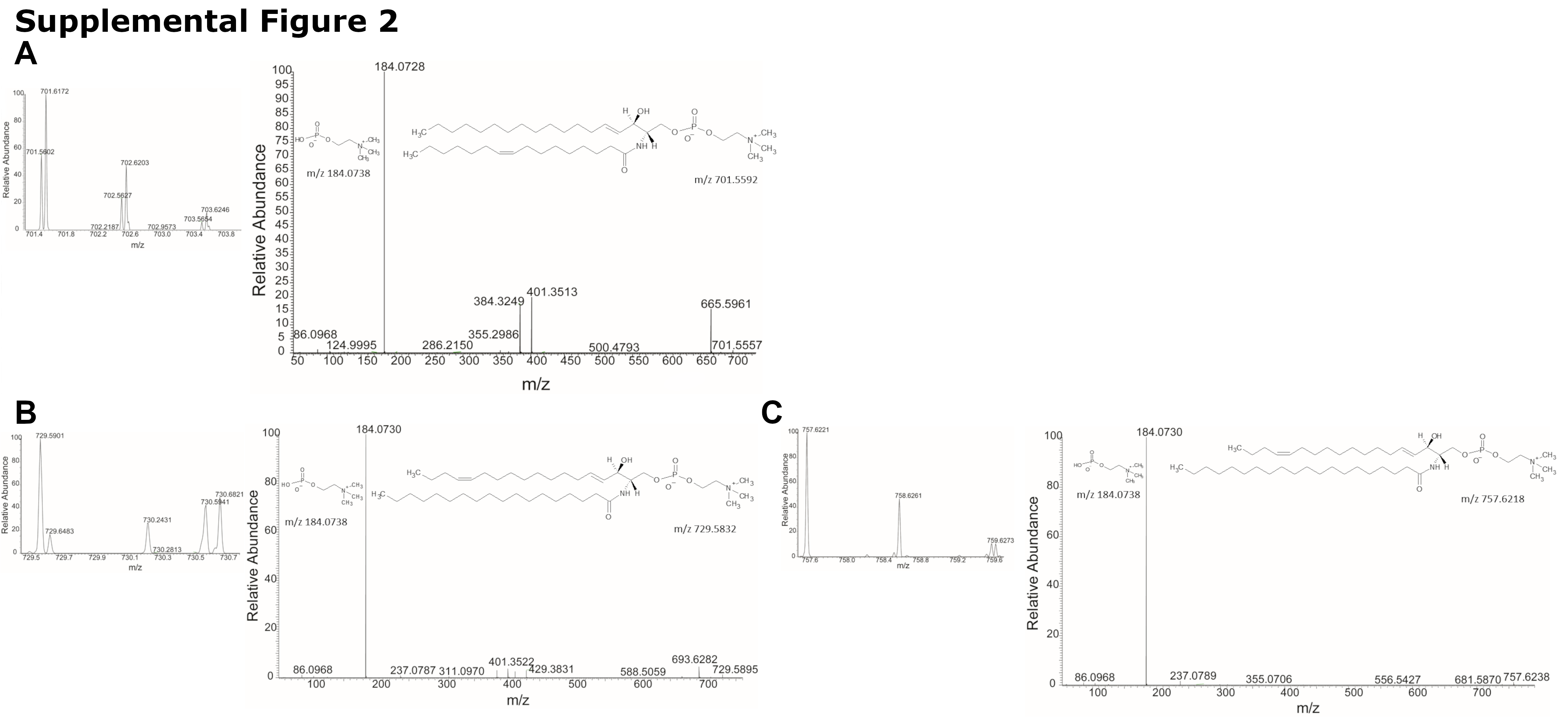

Supplement: FIG S2 [file msphere.00826-21-sf002.tif]

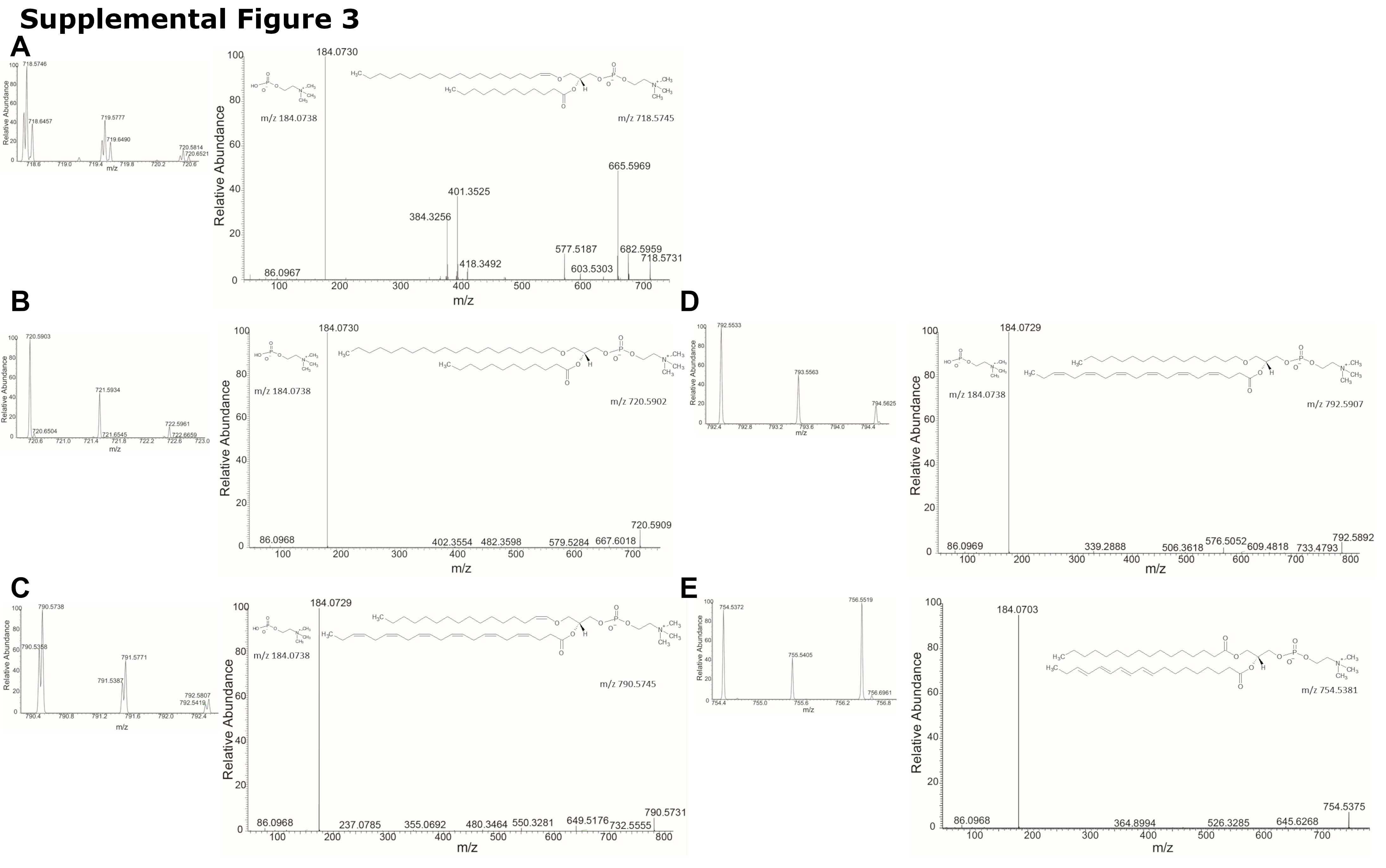

Supplement: FIG S3 [file msphere.00826-21-sf003.tif]

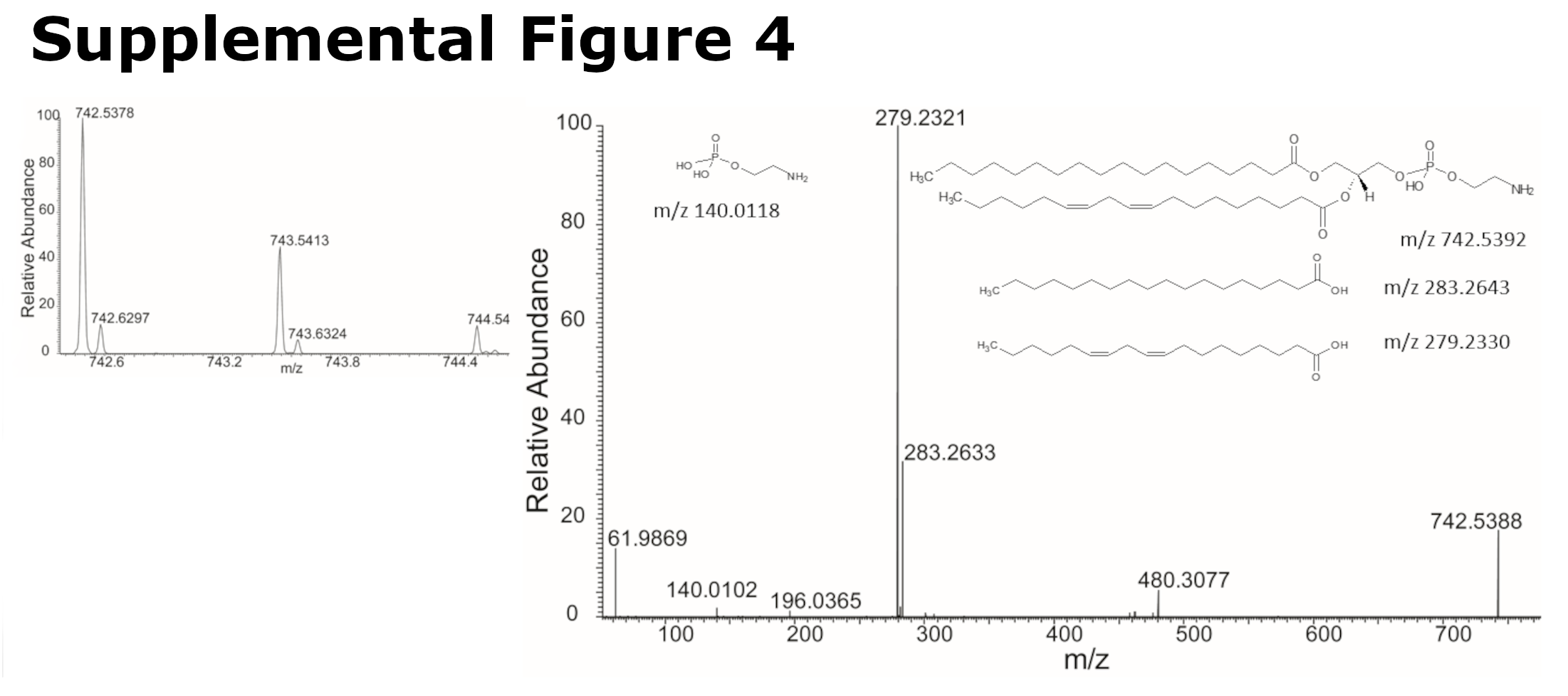

Supplement: FIG S4 [file msphere.00826-21-sf004.tif]

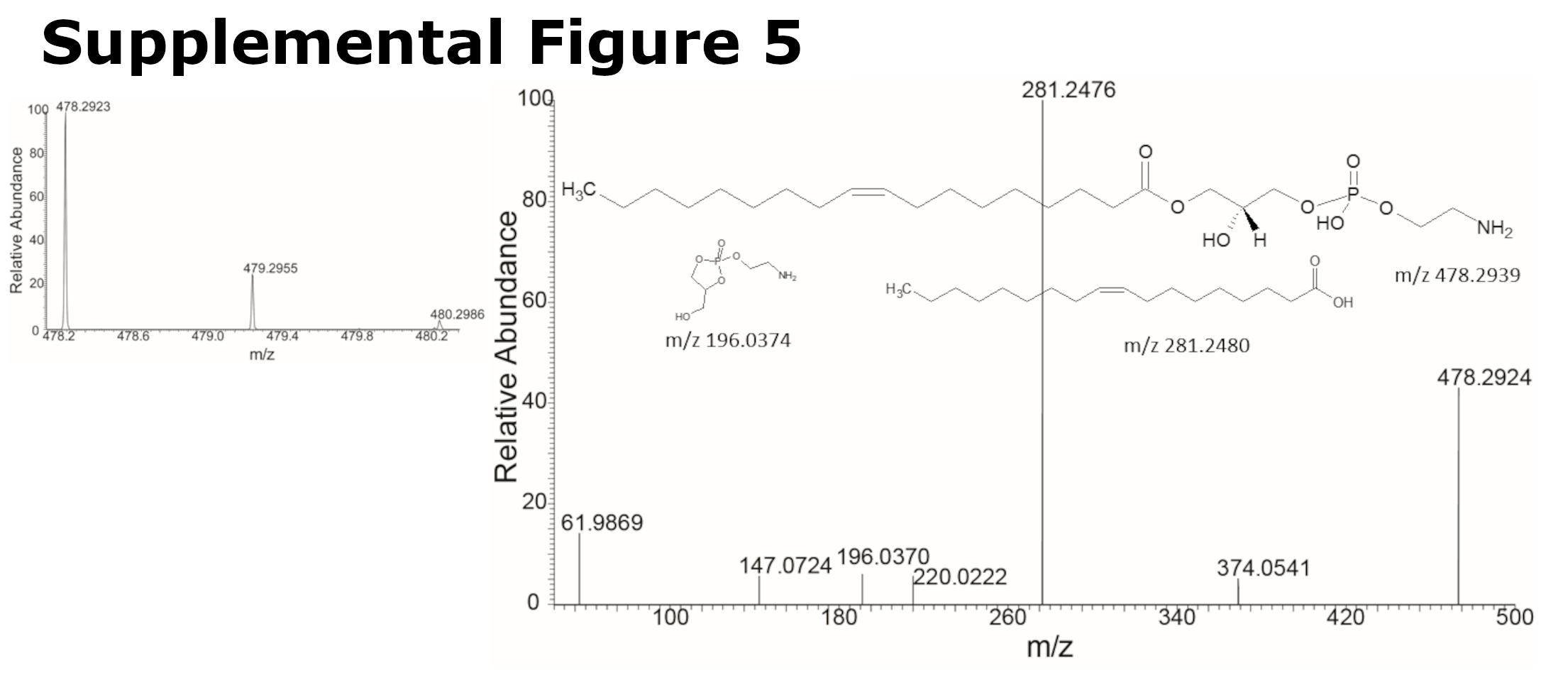

Supplement: FIG S5 [file msphere.00826-21-sf005.tif]

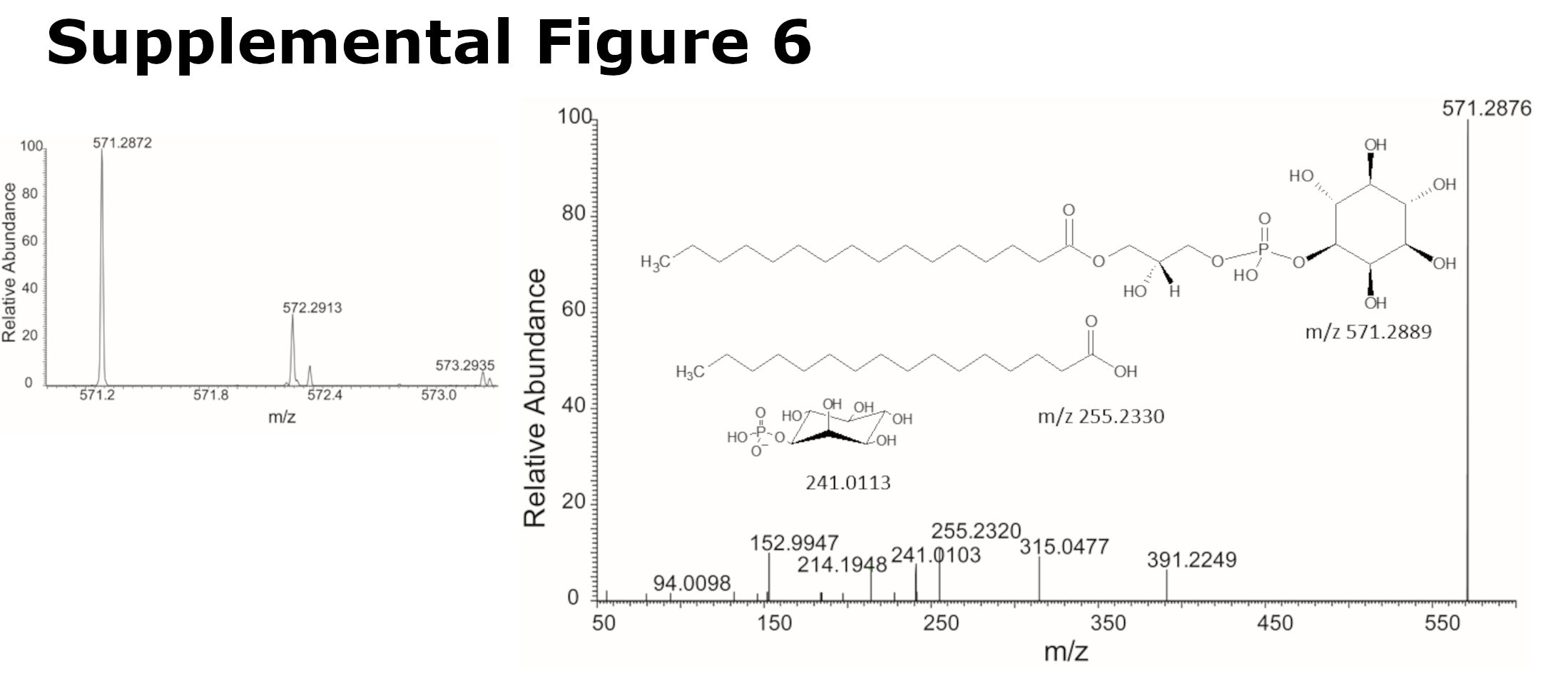

Supplement: FIG S6 [file msphere.00826-21-sf006.tif]

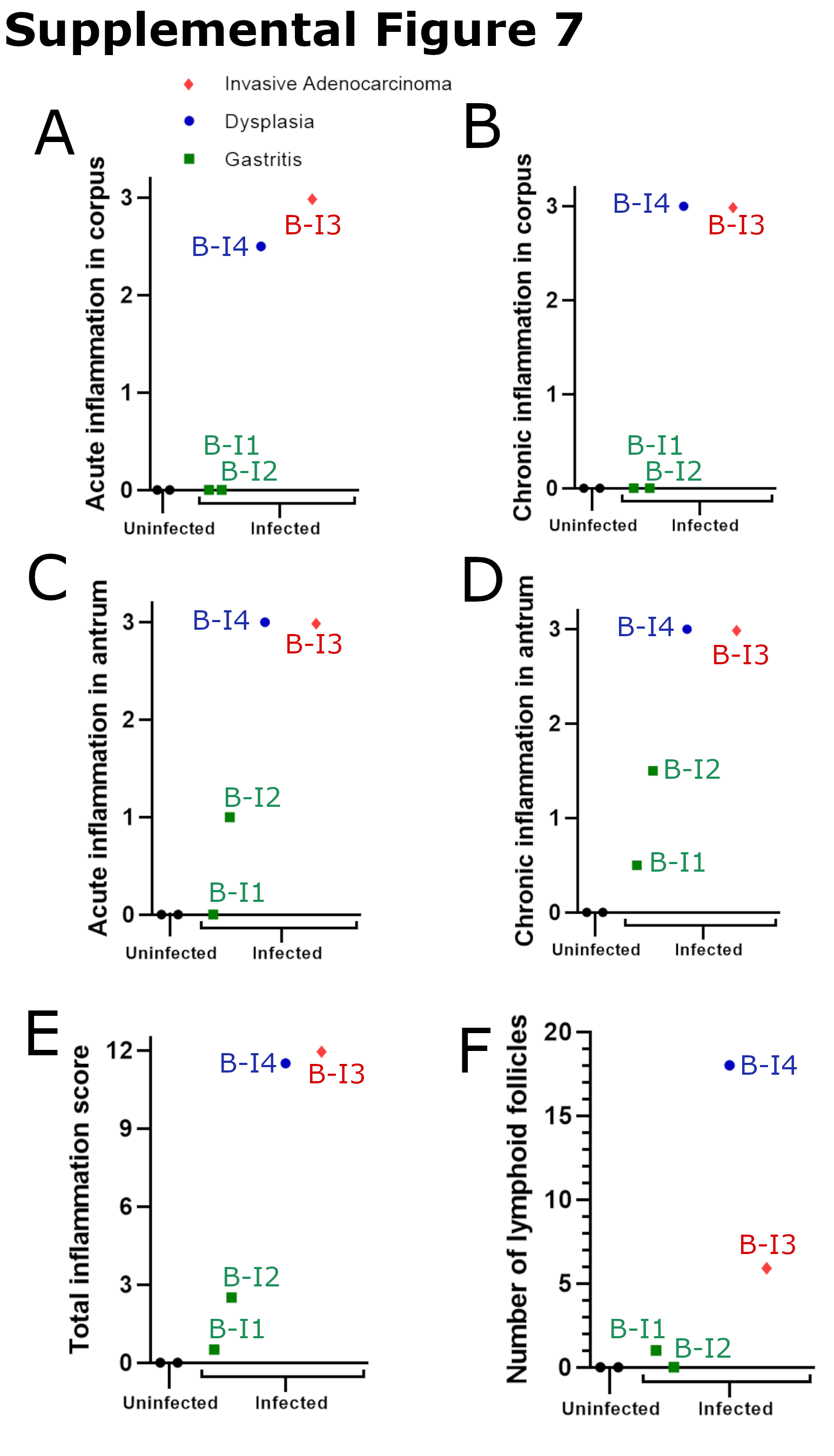

Supplement: FIG S7 [file msphere.00826-21-sf007.tif]
